# A Practical Platform for Blood Biomarker Study by Using Global Gene Expression Profiling of Peripheral Whole Blood

**DOI:** 10.1371/journal.pone.0005157

**Published:** 2009-04-17

**Authors:** Ze Tian, Nathan Palmer, Patrick Schmid, Hui Yao, Michal Galdzicki, Bonnie Berger, Erxi Wu, Isaac S. Kohane

**Affiliations:** 1 Informatics Program, Children's Hospital Boston, Harvard Medical School, Boston, Massachusetts, United States of America; 2 Division of Health Sciences and Technology, Harvard University and Massachusetts Institute of Technology, Cambridge, Massachusetts, United States of America; 3 Computer Science and Artificial Intelligence Laboratory, Massachusetts Institute of Technology, Cambridge, Massachusetts, United States of America; 4 Department of Mathematics, Massachusetts Institute of Technology, Cambridge, Massachusetts, United States of America; 5 Genomics Program, Children's Hospital Boston, Harvard Medical School, Boston, Massachusetts, United States of America; 6 Department of Pharmaceutical Sciences, North Dakota State University, Fargo, North Dakota, United States of America; Duke University, United States of America

## Abstract

**Background:**

Although microarray technology has become the most common method for studying global gene expression, a plethora of technical factors across the experiment contribute to the variable of genome gene expression profiling using peripheral whole blood. A practical platform needs to be established in order to obtain reliable and reproducible data to meet clinical requirements for biomarker study.

**Methods and Findings:**

We applied peripheral whole blood samples with globin reduction and performed genome-wide transcriptome analysis using Illumina BeadChips. Real-time PCR was subsequently used to evaluate the quality of array data and elucidate the mode in which hemoglobin interferes in gene expression profiling.

We demonstrated that, when applied in the context of standard microarray processing procedures, globin reduction results in a consistent and significant increase in the quality of beadarray data. When compared to their pre-globin reduction counterparts, post-globin reduction samples show improved detection statistics, lowered variance and increased sensitivity. More importantly, gender gene separation is remarkably clearer in post-globin reduction samples than in pre-globin reduction samples. Our study suggests that the poor data obtained from pre-globin reduction samples is the result of the high concentration of hemoglobin derived from red blood cells either interfering with target mRNA binding or giving the pseudo binding background signal.

**Conclusion:**

We therefore recommend the combination of performing globin mRNA reduction in peripheral whole blood samples and hybridizing on Illumina BeadChips as the practical approach for biomarker study.

## Introduction

Peripheral blood has recently become an attractive prime tissue for biomarker detection because of its critical role in immune response, metabolism, and communication with cells, and extracellular matrices in almost all tissues and organs in the human body, as well as its being less invasive and the simplicity of sample collection[1], [2], [3].

Many different techniques are used to handle peripheral blood samples prior to RNA isolation based on the experimental design: PAXgene (whole blood), QIAamp (Platelets and White Blood Cells), and Ficoll and BD-CPT (Mononuclear cells). Several studies have been conducted to make a comparison between methods by examining their reproducibility, variance, and signal-to-noise ratios[4]. Each method has its unique advantages and disadvantages. The PAXgene Blood RNA system provides a way to stabilize RNA immediately after sample collection and makes it possible to store the samples for a relatively long time without compromising the RNA's integrity [4], [5], [6], [7]. This is very important for multiple-center clinical practice. However, a high degree of variability and low present call rates have been observed in data obtained from Affymetrix microarrays when the samples are prepared using this whole-blood RNA system. These poor results are thought to be the effects of an overabundance of hemoglobin, and, consequently, several globin reduction methods have been developed to solve this problem[8], [9], [10], [11]. Although globin reduction significantly improved whole genome gene expression profiles in Affymetrix arrays, and post-globin reduction samples could be successfully applied to Illumina bead arrays[9], less is known about the impact of globin on Illumina bead array, since no comparison has been made between pre-globin reduction and post-globin reduction gene expression profiles in this array.

The Illumina Sentrix human-6 v2 array uses gene-specific 50-mer probes attached to 3 µm beads with an average of 30 redundant features for each transcript, allowing six samples to be profiled per BeadChip simultaneously[12]. This multi-sample approach provides the possibility of higher throughput for large-scale clinical research. In order to realize the potential impact of over-abundant globin on Illumina high-throughput expression array in medical practice, a practical framework for clinical microarray-based studies must be established. To this end, we compared the data obtained by microbead array profiling of pre-globin reduction and post-globin reduction peripheral whole blood samples. Our data demonstrated the combination of performing globin reduction in peripheral whole-blood samples and hybridizing on Illumina BeadChips to be the practical approach for large-scale multi-center biomarker research.

## Results

### Globin mRNA reduction improves cRNA signal

No difference was seen between pre- and post-globin reduction samples when comparing RNA quality using a bioanalyzer. Pre-globin reduction samples looked to be of similar quality to post-globin reduction samples, with two sharp peaks of 18 s and 28 s RNA around 2000 bp and 4000 bp. However, the cRNA from pre-globin reduction samples showed different signals from standard cRNA. Hemoglobin showed an additional sharp peak on the top of cRNA Fluorescence absorbance curve in pre-globin reduction samples and the sharp peak disappeared in post-globin reduction samples. In addition, most of the pre-globin reduction samples only showed a band at 800 bp in cRNA electrophoresis figure and post-globin reduction samples exhibited smeared bands (200 bp–6000 bp) as normal standard cRNA (Data not shown).

### Globin reduction improves microarray probe detection and decreases variance

A great deal of evidence shows that, when compared to other techniques, whole blood samples prepared by the PAXgene method typically result in Affymetrix microarray data with a low rate of genes detected as “present” with respect to the background noise on the chip, as well as large intra-group variance. The great abundance of hemoglobin mRNA is thought to account for this poor performance[13]. In order to quantify these observations, we analyzed the performance of the Illumina Sentrix human-6 BeadChip microarray platform on pre- and post-globin reduction peripheral whole-blood samples of 11 adults, 8 females and 3 males.

The BeadStudio software used to process Illumina's BeadChip data provides a “detection p-value” that can be used to determine whether a particular probe was detected against background noise. After correcting for multiple rounds of hypothesis testing, we considered adjusted p-values below 0.05 to be “detected” or “present.”

**Figure 1:**
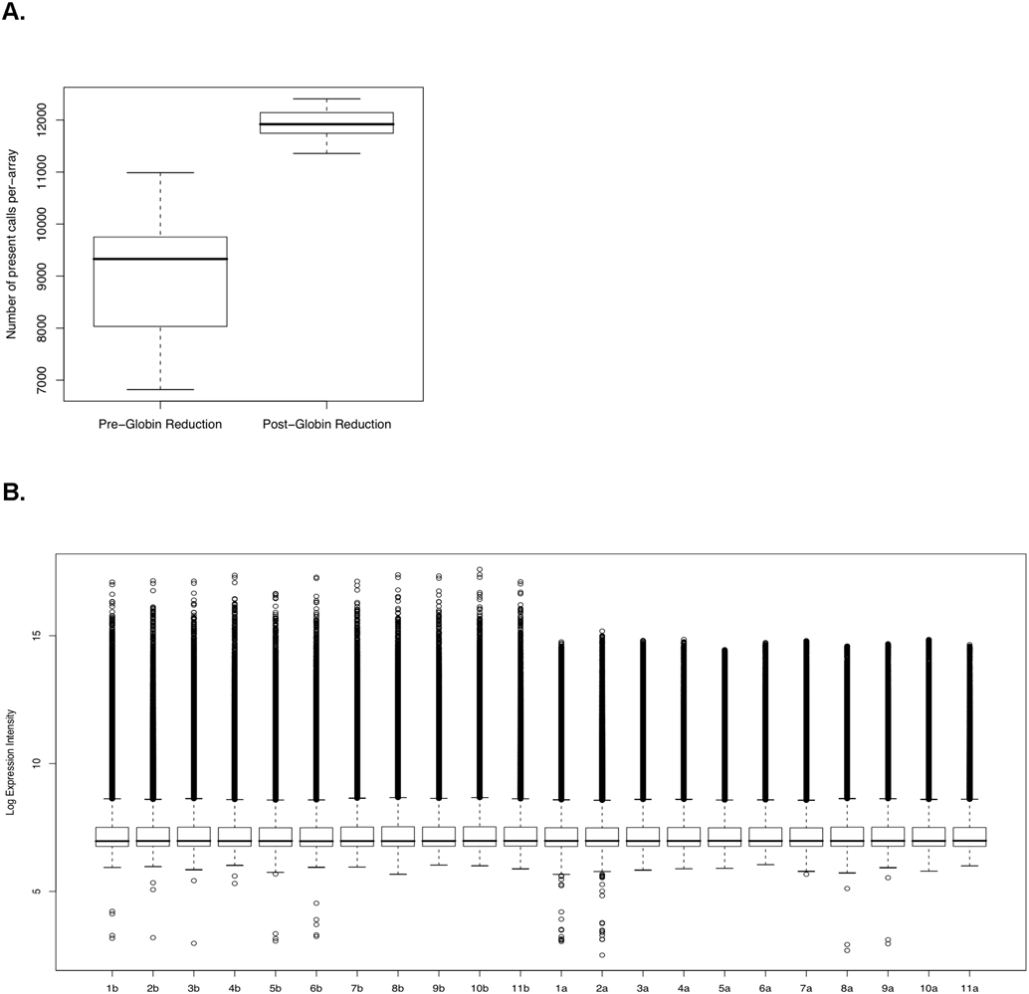
Globin reduction Increased present calls and decreased variance. Box plots showing the distribution of number of present calls per array in both the pre- and post-globin reduction data. Whiskers on the plots extend from the minimum and maximum values to the lower and upper quartiles, respectively. The box extends from the lower quartile through the upper quartile, with a bold line marking the median. B. Decreased intra-sample variance in intensity by globin reduction. Each column shows the distribution of log expression intensities for one sample as reported in the microarray data. Pre-globin reduction samples are labeled 1b–11b, and post-globin reduction samples are labeled as 1a–11a.

Given the above definition, the average number of present calls per array in the post-globin reduction group was 11921.73±296.38, whereas the average number of present calls per array in the pre-globin reduction group was significantly lower and more variable, at 8987.75±1264.94. Hence, samples in the post-globin reduction group showed improved probe detection and reduced intra-group detection call variance with respect to those in the pre-globin reduction group (Figure 1A). In addition, intra-sample intensity variance was reduced in the post-globin reduction group (Figure 1B).

In order to determine whether the improvement in detection was a consistent phenomenon (i.e., possibly probe- or target-dependent), we sought to identify probes that were consistently detected in the post-globin reduction group, and consistently *not* called in the pre-globin reduction group. Such probes might represent nucleotide sequence-specific susceptibility to globin-induced noise, or some other systemic effect.

1876 probes showed a consistent pattern of improved detection, where at least 75% of the adjusted detection p-values were <0.05 for the post-globin reduction group and at least 75% were >0.05 in the pre-globin reduction group with a statistically significant difference between the distributions of pre- and post-globin reduction adjusted p-values (Table S1). Here, statistical significance of the separation of sampling distributions was determined by computing the p-value of a Wilcoxon Mann-Whitney test between the pre- and post-globin reduction distributions, and thresholding at 0.05.

In addition, no genes were found to pass the detection criteria described above (75%<0.05 adjusted p-value) in the pre-globin reduction group, but failed to pass the criteria in the post-globin reduction group.

In order to show that the 1876 probes with consistently improved p-values are not an artifact of random fluctuation of the p-values reported by the BeadStudio software, a randomized simulation was run to estimate the number of probes that would pass our selection criteria under random re-association of improved detection calls to probe identifiers. After 100 random trials, none of the simulations produced more than one probe that passed our criteria for being significantly improved by globin reduction. These results indicate that the 1876 probes identified here were not an artifact of the stochastic nature of microarray hybridization and instead represent a set of probes whose improved detection was strongly dependent on the removal of globin RNA from the whole blood samples.

### Globin reduction improves the sensitivity of microarray experiments

The sensitivity threshold of microarray measurements defines the concentration range in which accurate measurements can be achieved. One of the advantages of the BeadArray platform is that it requires less mRNA for hybridization. This makes BeadArray more sensitive than any other platforms.

**Figure 2:**
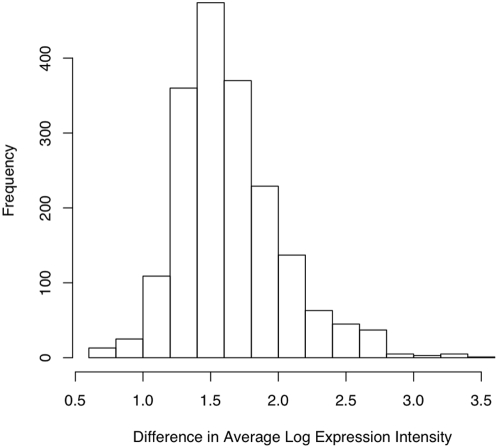
Probes with improved detection have higher expression intensity in post-globin reduction data compared to pre-globin reduction. The histogram shows the distribution of the difference in average log-reduced intensities between the post-globin reduction and pre-globin reduction data for the probes with improved detection p-values. Most of the improved probes show at least two-fold increase in average expression intensity in the post-globin reduction data.

**Figure 3:**
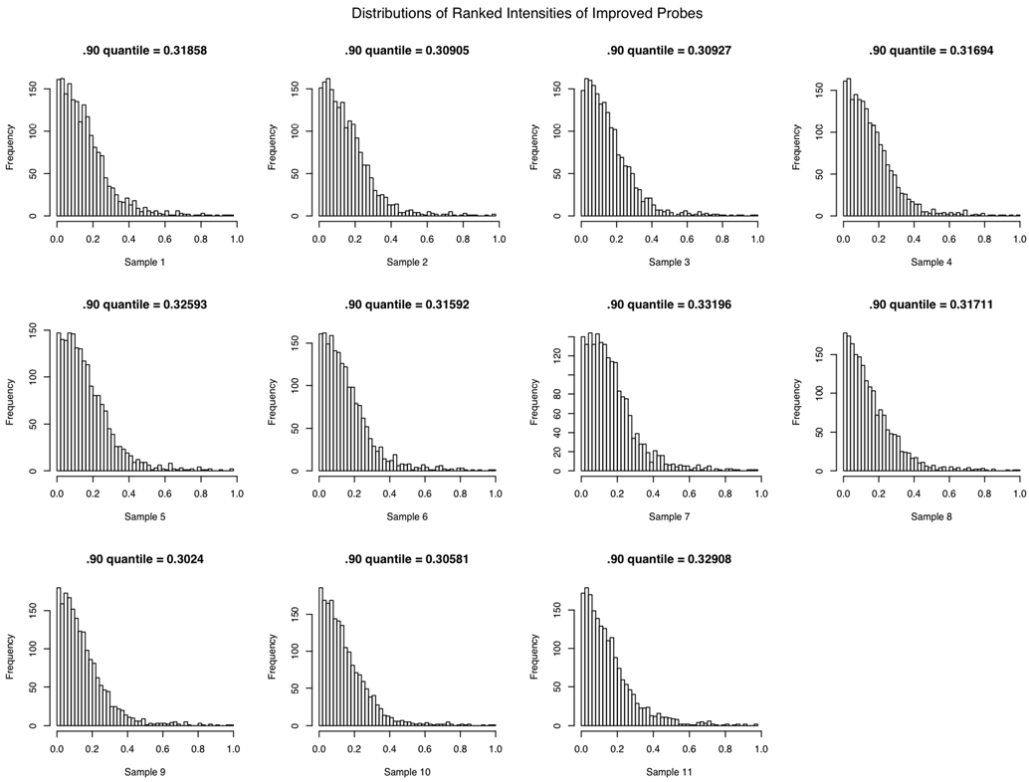
Globin reduction improved sensitivity. Probes with Improved Detection Values have Low Intensity. Each histogram shows the distribution of the ranks of the expression levels of the probes with improved detection p-values in the post-globin reduction data. The X-axis represents the ranks of expression level of the genes and Y-axis represents the frequency with which a given intensity was observed. All 11 samples show a clear tendency for the improved probes to have low expression level.

All of the 1876 genes with consistently improved detection p-values exhibited higher expression intensity in the post-globin reduction group than in the pre-globin reduction group (Figure 2). The BeadStudio software computes signal intensity by subtracting away background; this may be an indication that the improvement in detection is mainly due to reduced background noise caused by the over-abundance of globin mRNA. Moreover, in all of the 11 post-globin reduction samples, at least 90% of the probes with consistently improved detection values had expression intensities that were among the lowest 1/3 of all detected probes, i.e. low abundance genes (Figure 3). Thus, globin reduction improved detection sensitivity most dramatically for low abundance genes. This again supports the hypothesis that the improvement in detection is predominantly due to lower background noise induced by the overabundance of globin.

### Globin reduction improves useful biological signals

In order to show that the post-globin reduction improves the practical usability of whole blood mRNA samples, the pre- and post-globin reduction samples were used to select gender marker genes. A similar approach was used by Debey et al[9]. After performing the normalization and gene selection process described in the Materials and Methods section, we found that a significant fraction of the marker genes were in fact genes that are located on either the X or Y chromosome.

**Figure 4:**
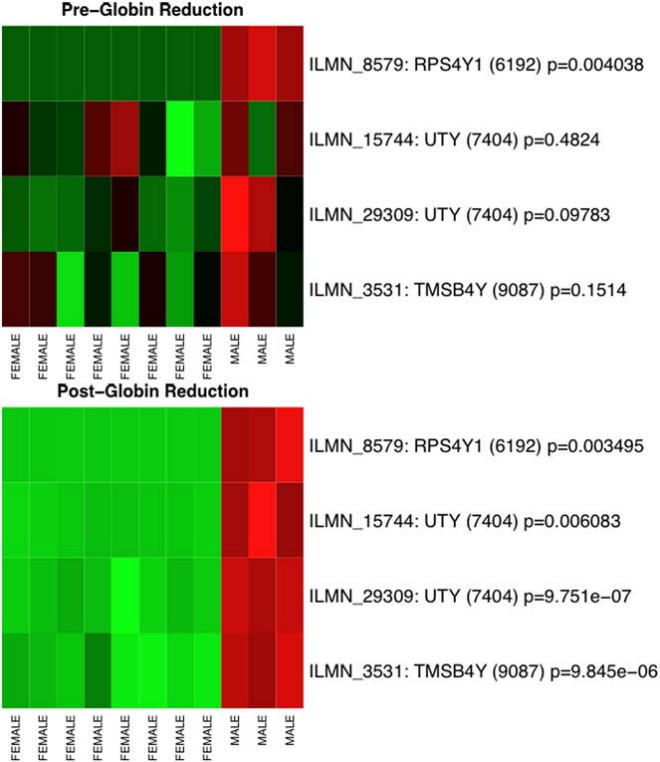
Globin reduction improves class separation. The panel on the top shows a heatmap of the marker genes in the pre-globin reduction data, with gender labels for the experiments on the columns and gene identifiers on the rows. Student's t-test p-values are also shown on the rows, describing the statistical separation between the male and female intensity distributions. The panel on the bottom shows a heatmap of the same marker genes in the post-globin reduction data. The post-globin reduction data shows a clear improvement in biological signal. These genes were identified, as described in Materials and Methods, by repeated ranking for discriminatory power based on the ReliefF algorithm.

The result of the selection process shows a clear performance improvement when finding gender marker genes using post-globin reduction samples instead of pre-globin reduction samples. For example, when the top ten probes are selected in each of the ten iterations using the pre-globin reduction data, only one was selected in at least half of the iterations. In contrast, when using the post-globin reduction experiments four probes, representing three distinct genes on the Y chromosome, were selected. Figure 4 shows heatmaps of the intensity values of these four selected probes in the pre- and post-globin reduction groups. The separation between the male and female samples was much more clearly defined in the post-globin reduction samples. Similarly, when selecting the top 40 probes from the untreated experiments only nine were found. Of those, only five were on the X or Y chromosome. When using the post-globin data, however, 12 probes were found, 10 of which were on either the X or Y chromosome. With only one exception, the p-values from a Mann-Whitney U test for the male-to-female comparison in the post-globin samples were more significant than those from the pre-globin samples. The one exception is likely the result of small sample size. Thus, in the context of biomarker selection, the post-globin reduction samples yield not only more statistically significant results than the pre-globin reduction samples, but also pick out more biomarkers with higher accuracy.

### Evaluation of the differentially expressed genes by real time PCR

As well as improving the detection of low intensity and biologically informative transcripts, the globin reduction treatment also resulted in 298 genes (all with strong detection calls in both treatment groups) exhibiting apparent differential expression between the pre- and post-globin reduction groups. Among these, 75 genes were up-regulated with fold changes ≧2; 223 genes were found down-regulated with fold changes ≤0.5. We postulate first that hemoglobin may compete with the target mRNA in binding to the probe, thus contributing to a higher-than-normal pseudo binding background signal. Second, the overabundance of hemoglobin may interfere with target mRNA binding through an unknown mechanism.

**Figure 5:**
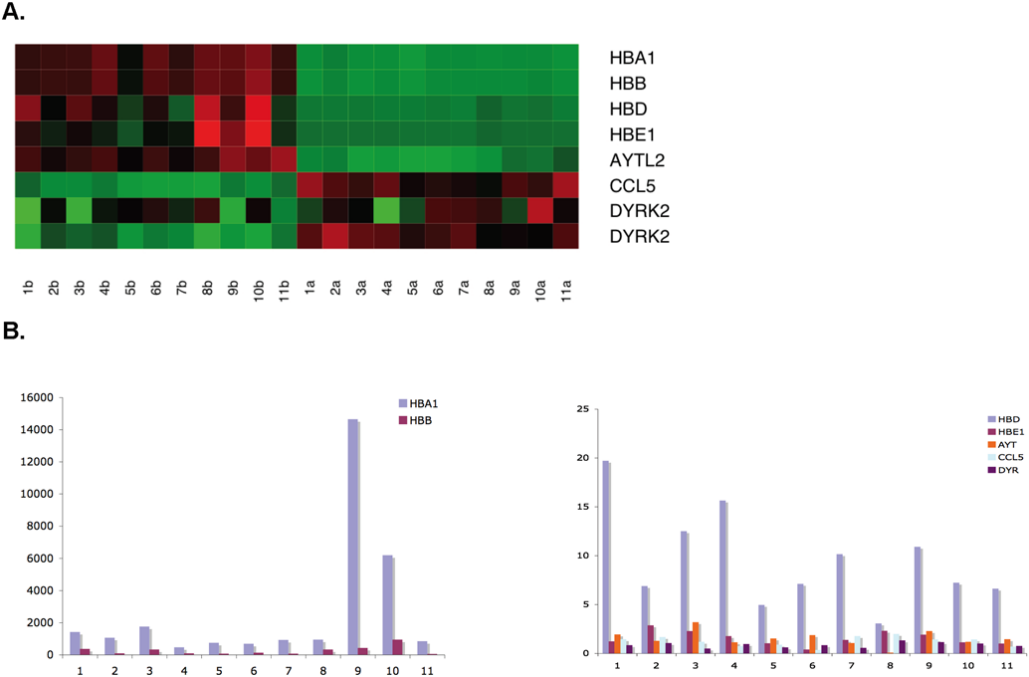
Real-time PCR evaluations of genes identified as differentially expressed between pre- and post-globin reduction samples in the microarray data. A. A heatmap showing seven of the genes in 11 samples differentially expressed between the pre- and post-globin reduction samples in the BeadArray data. B. The four hemoglobin genes were significantly reduced and no other gene was markedly changed by globin reduction in real-time PCR data. The X-axis represents 11 samples and Y-axis represents the gene expression ratio of pre-globin reduction to post-globin reduction.

We used real-time PCR to show that the RNA levels of these genes were, in fact, no different in the pre- and post-globin reduction samples, and that the differential expression observed in the array data was an artifact of the noise induced by the overabundance of globin RNA in the pre-globin reduction samples. Four hemoglobin genes, HBA1, HBB, HBD, and HBE1, together with another down-regulated gene, as well as 2 up-regulated genes in bead array were randomly chosen to verify by real-time PCR. The two most abundant hemoglobin genes, HBA1 and HBB, were observed at lower levels in the post-globin reduction group with respect to the pre-globin reduction group in both the array data and in the real time PCR, indicating that the globin reduction protocol was effective. Two other hemoglobin genes, HBD and HBE, were also significantly down-regulated by globin reduction in both assays. However, AYTL2, the gene observed to be down-regulated in the array data, and the 2 genes that showed higher expression levels in the array data, ccl5 and DYRK2, all showed no significant change in expression level in the real-time PCR assay (Figure 5).

## Discussion

Expression profiling using peripheral whole blood samples is an attractive method for biomarker detection. However, hemoglobin, which represents as much as 70% of the total mRNA population in peripheral whole blood samples isolated by the PAXgene tube, effectively dilutes the mRNA population and interrupts the gene expression profiles in the Affymetrix array[9]. It is of interest to investigate whether and how hemoglobin influences the gene expression profiles acquired from Illumina bead arrays, which constitute a high throughput platform. In this study, we compared the gene expression profile of 11 pre- and post-globin reduction peripheral whole blood samples hybridized on Illumina bead arrays. We demonstrated that hemoglobin influenced the gene expression profiles from these arrays in a clear and consistent manner. Globin reduction efficiently improved the probe detection by increasing present calls and decreasing variance, as well as improving sensitivity of lower abundance genes. More importantly, the more consistent expression signature of 4 sex genes in the post-globin reduction group with respect to the pre-globin reduction group indicates that class prediction was markedly improved with globin reduction. We reasoned that the high abundance of hemoglobin might interrupt the target mRNA binding, or contribute to pseudo-binding (a nonspecific, background signal that is present in the absence of any significant sequence similarity) to the probe, and, therefore, distort the true expression signal.

Real-time PCR is commonly used to validate the mRNA expressions acquired from microarray experiments due to the greater specificity of the primer vs. microarray probes[14]. Therefore, real-time PCR was used as a “truth” measurement to evaluate the reliability of the pre- and post-globin reduction bead array data. Four hemoglobin genes, HBA1, HBB, HBD and HBE1, along with 3 randomly selected differentially expressed genes (one down-regulated and two up-regulated by globin reduction), were chosen for mRNA level measurement by real time PCR. We found that, although the GLOBINclear kit is claimed to only reduce HBA1 and HBB, the other hemoglobin genes HBD and HBE1 also showed significantly lower mRNA levels in the post-globin reduction samples when compared to the pre-globin reduction samples. This might be due to the fact that HBD and HBE1 have 93% and 79% percent sequence identify with HBB (http://www.ncbi.nlm.nih.gov/blast/Blast.cgi), respectively. However, the non-hemoglobin gene that was observed to be down-regulated after globin reduction in the microarray data, AYTL2, showed no significant change in the real-time PCR data. This suggests that globin reduction did not actually decrease the level of other genes' mRNA, but rather allowed for more accurate measurement of these levels when the samples were hybridized to microarrays. The decreased intensity observed for non-hemoglobin genes on the post-globin reduction microarrays might be due to high abundance hemoglobin providing a non-specific pseudo-binding signal. On further analysis, no significant sequence similarity was found between these down regulated genes and HBA1 and HBB. This again supports our hypothesis that the higher expression in pre-globin reduction samples of some genes might be due to a non-specific pseudo-binding signals from hemoglobin. In addition, the two up-regulated genes (ccl5 and DYR) exhibited no significant changes in expression level in the real time PCR data. This *indicates* that hemoglobin may *interfere with target mRNA binding through an unknown mechanism and result* in lower expression signals from the pre-globin reduction microarrays.

In summary, this study d*emonstrates that* the combination of performing globin mRNA reduction in peripheral whole blood samples and hybridizing on Illumina BeadChips is a practical approach for biomarker research. Our future study will focus on the cancer biomarker detection by using this established platform.

## Materials and Methods

### Sample collection and RNA preparation

This study was conducted under protocols approved by the Children's Hospital Boston Institutional Review Board. Blood samples were obtained from 11 subjects who voluntarily agreed to participate and gave written informed consent. Peripheral blood was drawn with a BD safely Lok™ blood collection set (BD, Franklin Lakes, NJ) into PAXgene RNA collection tube (Qiagen, Valencia, CA) according to the standard procedure. Total RNA was prepared with the PAXgene Blood RNA Kit (Qiagen) according to the manufacturer's instructions with an on-column DNase digestion step. RNA quantity and quality were determined by a NanoDrop ND-1000 Spectrophotometer (NanoDrop Technologies, Wilmington DE) and an Experion™(Bio-RAD, Hercules, CA).

### Globin reduction

Since a large amount of hemoglobin exists in erythrocytes, several studies have shown decreased present calls, reduced accuracy, and increased variability among replicates in an Affymetrix GeneChip array when using PAXgene RNA collection technology[5], [13]. To overcome this obstacle, the GLOBINclear™ Kit (Ambion, Austin, Texas) was employed to remove the highly abundant hemoglobin mRNA according to the manufacturer's instructions. In short, 4 µg total RNA from each sample were hybridized with a biotinylated Capture OLIGO Mix that is specific for human mRNA hemoglobin α and β. Streptavidin Magnetic Beads were added to bind the biotinylated oligonucleotides that hybridized with globin mRNA and then were pulled down by magnet. The globin mRNA depleted RNA was transferred to a fresh tube and further purified with a rapid magnetic bead-based purification process.

### RNA amplification and hybridization on Illumina Sentrix humanref-6 arrays

100 ng of total RNA was applied to generate cRNA by using a Illumina TotalPrep RNA Amplification Kit (Ambion). Reverse transcription with the T7 oligo (dT) primer was used to produce first strand cDNA. The cDNA then underwent second strand synthesis and RNA degradation by DNA Polymerase and RNase H, followed by clean up. *In vitro* transcription (IVT) technology, along with biotin UTP, was employed to generate multiple copies of biotinylated cRNA The labeled cRNA was purified via Filter Cartridge and quantified by NanoDrop and RiboGreen® (Molecular Probes Inc. Eugene, OR). The integrity of cRNA was evaluated using an Experion™ (Bio-RAD).

The labeled cRNA target (1.5 µg) was used for hybridization to an array according to the Illumina Sentrix humanref-6 beadchip protocol. A maximum of 10 µl cRNA was mixed with a 20 µL GEX-HYB hybridization solution. The preheated 30 µl assay sample was dispensed onto the large sample port of each array and incubated for 18 hours at 58°C at a rocker speed of 5. Following hybridization, the samples were washed according to the protocol and scanned with a BeadArray Reader (Illumina, San Diego, CA).

### Real-time PCR

Hemoglobin α (HBA1), β (HBB), δ (HBD), and ε(HBE1) genes together with 3 other randomly chosen genes from a differential expression list were picked for evaluating the array data by using real-time PCR. Primers were designed using primer 3 and shown in Table 1. Briefly, 1 µg RNA of each sample was used for cDNA synthesis following the protocol described in the iScript cDNA synthesis kit (Bio-RAD). Real Time PCR was performed on the iQ5 Real-Time PCR detection system with the iQ SYBR Green Supermix (Bio-RAD) and GAPDH was used as an internal control. The relative quantification of mRNA expression was calculated according to the literatures[15], [16], [17].

**Table 1:** Primers of selected genes for real-time PCR.

Gene	Forward	Reverse
HBA1	ACGGCTCTGCCCAGGTTAAG	GTATTTGGAGGTCAGCACG
HBB	GCAACCTCAAACAGACACCA	CAGCATCAGGAGTGGACAGA
HBD	GGAGGACAGGACCAGCATAA	CAGATCCCCAAAGGACTCAA
HBE1	TGGAAACCTGTCGTCTCC	TTGCCAAAGTGAGTAGCC
AYTL2	GGTGTGAACTCAAGGGCCTA	TATCCAACCTCGGACTGGAG
DYRK2	CTCACGGACAGATCCAGGTT	TGCTTCATTGCTTGTTCAGG
CCL5	CGCTGTCATCCTCATTGCTA	ACACACTTGGCGGTTCTTTC
GAPDH	GAGTCAACGGATTTGGTCGT	TTGATTTTGGAGGGATCTCG

### Data extraction and statistics

Detection p-values produced by the BeadStudio software were corrected for multiple hypothesis testing[18]. The R software package[19] was used for statistical analysis, as were several components of the BioConductor[20] libraries for R. The Wilcoxon Mann-Whitney test was used to identify probes with a statistically significant separation of adjusted p-values between the pre- and post-globin reduction groups. Gene selection was performed independently on the pre- and post- globin reduction groups using the ReliefF algorithm[21], [22], [23] as implemented in the WEKA machine learning package[24].

In order to show that the probes with consistently improved detection p-values were not an artifact of random chance, a randomized simulation was run. Each trial in the simulation consisted of identifying, for each blood sample (a pre- and post-globin reduction pair of microarray experiments), those probes with p-values greater than 0.05 in the pre-globin reduction data and less than 0.05 in the post-globin reduction data. Those p-values were then randomly reassigned among the probes whose p-values were not below 0.05 in both the pre- and post-globin reduction data. After performing this random re-association for each blood sample, we applied the same criteria for selecting significantly improved probes as was applied to the observed data, and recorded the number of probes that passed the selection criteria. Thus, our simulation generates a distribution according to the null hypothesis that an improved p-value pair is equally likely to be associated with any probe whose p-values are not already below 0.05 in both the pre- and post-globin reduction samples.

To verify the efficacy of the globin reduction treatment in the context of gene selection, the following normalization and selection process was run. Analysis of the raw intensity data revealed that, between pairs of arrays, a non-linear relationship existed between corresponding pairs of probes. To correct this, we used a Loess adjustment as implemented in the BioConductor package for R [20]. Loess normalization is a standard microarray normalization method that removes non-linear intensity-dependent artifacts from the data by iteratively fitting a series of local piecewise curves to the log-mean-difference plots of each pair of arrays, and effectively subtracting the curve from the data[25].

After normalization, the data was split into pre- and post-globin reduction groups, each containing eight female and three male samples. We then performed gene selection independently on these two groups of 11 experiments using the ReliefF algorithm[21], [22], [23], [24]. Our goal when identifying marker genes was to find genes that are best able to separate male samples from female samples, and to determine whether the results were more reproducible in the post-globin reduction data than in the pre-globin reduction data. Due to the small sample size and unequal number of male and female subjects, the gene selection process was repeated ten times on a subset of the data. Each repeat used three random female samples and compared them against all three male samples. To perform the gene selection in each repeat, we used the ReliefF algorithm. ReliefF ranks the individual genes by their ability to distinguish gender based on intensity values. Briefly, for each experiment *e*, the ReliefF algorithm finds *e*'s nearest neighbor with the same class (gender) using Euclidian distance over all genes. The nearest neighbor of the other class (opposite gender) is also found in the same manner. These selections are called the hit and miss, respectively. The importance of each gene is then computed by taking a normalized sum of differences between the distance from *e* to the hit and the distance from *e* to the miss. Thus, the greater the difference between the hit and miss, the greater the importance of the gene in distinguishing class. This method has the advantage that it makes no assumptions about the distribution of expression intensities. Marker genes were identified by picking out genes that were consistently ranked highly across at least half of the repeats. Heatmaps, as well as Student's t-test p-values describing the ability of the genes identified by the above method to distinguish gender were generated in R.

## Supporting Information

Table S1 (0.52 MB XLS)
